# Evolution of rural–urban health gaps in Morocco: 1992–2011

**DOI:** 10.1186/1756-0500-5-381

**Published:** 2012-07-27

**Authors:** Boutayeb Abdesslam

**Affiliations:** 1 LaMSD, Faculty of Sciences, University Mohamed Ier, Unité Associée au CNRST URAC04, Oujda, Morocco

**Keywords:** Health equity, Gender, Rural, Urban, Indicators, Differences, Maternal, Infant

## Abstract

**Background:**

Moroccan authorities carry out regular surveys on population and family health (NSFFP 1980, NSPH 1992, SPFH 2004, NSPFH 2011). These surveys constitute valuable resources for monitoring socio-economic and health indicators. They provide an evidence base for health decision makers to help them to optimize health strategies in order to improve the health conditions of the whole population. They also provide updated measures on geographic disparities, socio-economic inequalities and health inequity. The most recent Moroccan population and family health survey (NSPFH 2011) was carried out between November 2010 and March 2011. The final report and the database are not yet accessible, but a preliminary report was released early March 2012. This report does not allow for a complete evaluation of the present health situation in Morocco. A partial equity analysis can, however, be devoted to the comparison of health indicators especially in terms of rural–urban gaps.

**Results:**

The 2011 survey shows that Moroccan population is in the last phase of the demographic transition. The total fertility rate decreased from 5.6 children per woman in 1980 to 2.5 *per* woman in 2011. The mean age of first marriage increased from 24 years for men and 17.5 years for women in 1960 to 31.5 years and 26.3 years in 2011 for men and women, respectively. The age structure shows a trend of ageing population. A comparison with the 1992 NSPH indicates that adult illiteracy has decreased from 53% in 1992 to 37.6% in 2011.

During the same time period, women’s access to maternal care and health services improved significantly. For instance, the proportion of deliveries assisted by skilled health personnel increased from 31% in 1992 to 73.6% in 2011. Between 1992 and 2011, neonatal, postnatal, infant and under-five mortality rates were reduced by 44%, 65%, 54% and 64%, respectively.

**Conclusion:**

This paper shows that average health indicators improved noticeably during the last two decades but rural–urban disparities are still a challenge for health decision makers. Socio-economic indicators, like illiteracy rate and unemployment, also demonstrate large gender inequalities. This preliminary analysis is designed to assist Moroccan health authorities to evaluate the current health situation in order to adopt cost-effective strategies that improve “health for all” and reduce the gaps between advantaged and disadvantaged populations.

## Background

In addition to the general censuses of population (1960, 1972, 1982, 1994, 2004) [1], Moroccan authorities carry out regular surveys of population and family health (NSFFP 1980, NSPH 1992, SPFH 2004, NSPFH 2011) [2,3]. These surveys constitute valuable resources for monitoring socio-economic and health indicators. They allow health decision makers to adjust and optimize health strategies in order to improve health conditions of the whole population (on average) and they also offer updated measures on geographic disparities, socio-economic inequalities and health inequity. The last National Survey on Population and Family Health (NSPFH 2011) was carried out from November 2010 to March 2011. The final report and the whole database are not yet accessible but a preliminary report was released in March 2012 [3]. The information given so far does not allow a complete analysis and evaluation of the present health situation in Morocco to be made, compared to data provided by the previous surveys. The available data are, however, sufficient for a partial equity analysis especially in terms of rural–urban gaps.

Since the last report released by the WHO Commission on Social Determinants of Health in 2008 [4], and the Rio Political Declaration on Social Determinants of health adopted by heads of government, ministers and government representatives in October 2011 [5], health equity should become part of a government’s social and health agenda. It is to be a shared responsibility requiring the engagement of all sectors of governments, and all national and international agencies in “an all-for-equity” global action. This preliminary analysis is designed to assist Moroccan health authorities to evaluate the current health situation in order to adopt cost-effective strategies that improve “health for all” and reduce the gaps between advantaged and disadvantaged populations.

## Method

### Data collection

The National Survey on Population and Family Health (NSPFH 2011) is a three-stage stratified cluster-random sample of 15,577 households (75061 individuals). A total of 15,343 households were investigated, with a response rate of 98.5%. This high response rate can be explained by the fact that all households forming the sample were investigated and respondents were approached in person. Following the standard questionnaires of Pan Arab Family Health Surveys (PAPFAM), a household questionnaire included items on household characteristics, general health, housing conditions and anthropometric data for children less than six years of age. A second questionnaire specifically for women requested information on their resources, marriage, reproductive health, family planning, healthcare and nutrition, and knowledge of AIDS/SIDA.

### Analysis

This paper relies on the partial data released by the Ministry of Health in a preliminary report; the full data base is not yet accessible. Absolute differences and relative ratios were used to study the evolution of rural–urban gaps and gender differences in terms of literacy and employment, using health and socio-economic indicators. Unfortunately, with the limited data at hand, it was not possible to carry out a complete statistical analysis, using tests, confidence intervals and elaborate data analysis.

## Results

### Demographic indicators

In addition to current economic and political transitions, Morocco is undergoing a demographic transition. The population is now predominantly urban rather than rural, and the annual growth rate has decreased from 2.8% during the decade 1960–1970 to 1% between 2004 and 2011. The mean age of first marriage has increased from 24 years for men and 17.5 years for women in 1960 to 31.4 years and 26.6 years in 2011 for men and women respectively (Table 1).

**Table 1:** **Demographic and urban/rural evolution of the Moroccan population according to general censuses from 1960 to 2004 and estimation for 2011 **[1]

**Year of**	**Population (millions)**					**Annual growth rate**	**Mean age at first marriage (years)**							
**census**	**Urban**	**(%)**	**Rural**	**(%)**	**Total**		**Urban**		**Rural**		**National**			
**(RGPH)**							**M**	**F**	**M**	**F**	**M**	**F**		
1960	3.4	(29)	8.2	(71)	11.6		24.4	17.5	23.8	17.2	24.0	17.5
1971	5.4	(35)	9.9	(65)	15.3	2.8%	26.0	21.0	24.0	18.5	25.0	19.3
1982	8.7	(43)	11.7	(57)	20.4	2.6%	28.5	24.0	25.6	21.0	27.2	22.3
1994	13.4	(51)	12.7	(49)	26.1	2.1%	31.2	26.9	28.3	24.2	30.0	25.8
2004	16.5	(55)	13.4	(45)	29.9	1.4%	32.2	27.1	29.5	25.5	31.2	26.3
2011	18.5	(58)	13.4	(42)	31.9	1.0%	32.5	27.4	30.0	25.6	31.4	26.3

The total fertility rate decreased from 5.6 children *per* woman in 1980 to 2.5 children *per* woman in 2004 but has remained at the same level during the last decade. Consequently, the age structure indicates a trend of an ageing population (Table 2).

**Table 2:** **Evolution of fertility and age structure of the Moroccan population participating in National Surveys from 1980 to 2011 **[2,3]

**Survey (year)**	**ENFPF (1980)**	**ENPS (1992)**	**EPSF (2004)**	**ENPSF (2011)**
Total fertility rate	5.6	4.0	2.5	2.5
Age structure				
0–14	43.6%	39.7%	32.0%	29.0%
15–64	52.7%	55.6%	62.7%	64.2%
65+	3.7%	4.6%	5.4%	6.8%

ENFPF : Enquête Nationale de Fécondité et de Planification Familiale (National Survey on Fecondity and Family Planning).

ENPS : Enquête Nationale sur la Population et la Santé (National Survey on Population and Health).

EPSF : Enquête sur la Population et la Santé Familiale (Survey on Population and Family Health).

ENPSF : Enquête Nationale sur la Population et la Santé Familiale (National Survey on Population and Family Health).

The country is also undergoing an epidemiological transition. For example, the proportion of population suffering from at least one chronic disease increased from 14.8% (F: 17.5%; M: 11.8%) in 2004 to 18.2% (F: 21.3%; M: 14.9%) in 2011.

### Socio-economic indicators

Morocco embarked upon the third millennium with nearly half of the population illiterate. Although adult illiteracy decreased from 53% in 1992 to 37.6% in 2011, this level is still a handicap for human development. Moreover, illiteracy is inequitably distributed, with rural populations having higher levels of illiteracy (60.5%) than their urban counterparts (29.4%) Illiteracy among women (54.7%) is nearly the double of that of men (30.8%) (Table 3).

**Table 3:** **Comparison of gender and rural–urban gaps in illiteracy for the Moroccan population participating in the National Surveys on Population and Health 1992, 2004 and 2011 **[2,3]

**Illiteracy 10 years +**	**Male**	**Female**	**Both sexes**	**Ratio F/M**
Year 1992				
Urban	23.3	42.2	32.7	**1.8**
Rural	58.3	82.5	70.5	**1.4**
National	41.9	63.9	53.0	**1.5**
Ratio R/U	**2.5**	**1.9**	**2.2**	
Year 2004				
Urban	18.8	39.5	29.4	**2.1**
Rural	46.0	74.5	60.5	**1.6**
National	30.8	54.7	43.0	**1.8**
Ratio R/U	**2.4**	**1.9**	**2.1**	
Year 2011				
Urban	15.5	34.7	25.4	**2.2**
Rural	40.0	66.2	53.6	**1.7**
National	26.0	48.4	37.6	**1.8**
Ratio R/U	**2.6**	**1.9**	**2.1**	

Currently, unemployment is a major concern worldwide. In Morocco, the 2011 survey indicates that among women aged 15 years and over, the unemployment rate reaches 81% in cities and 94% in rural areas, compared to 37% and 24% respectively for men.

### Maternal and infant health indicators

During the last two decades, Moroccan authorities have noticeably improved maternal health by improving access to health services and promoting family planning. The recent survey shows that 77% of Moroccan women received prenatal care, compared to only 21.5% in 1992. By 2011 the percentage of assisted deliveries and deliveries in health facilities also increased to reach 73.6% and 72.7% respectively, although differentials between rural and urban areas persist.

Finally, the proportion of women receiving postnatal care remains very low (21.9%) especially in rural areas (13.2%) (Table 4).

**Table 4:** **Percentages of women having access to health care for the Moroccan population participating in the National Surveys on Population and Health 1992, 2004 and 2011 **[2,3]

	**% of women who had prenatal care**	**% of deliveries in health services**	**% of assisted deliveries**	**% of women who had postnatal care**
Year 1992				
Urban	61.0	59.0	64.0	N/A
Rural	18.0	12.5	14.0	N/A
National	21.5	28.5	31.0	N/A
Ratio R/U	**3.4**	**4.7**	**4.6**	
Year 2004				
Urban	85.0	83.2	85.3	16.3
Rural	48.0	38.0	39.5	3.6
National	68.0	61.0	63.0	6.6
Ratio R/U	**1.8**	**2.2**	**2.2**	**4.5**
Year 2011				
Urban	91.6	90.7	92.1	30.5
Rural	62.7	54.6	55.0	13.3
National	77.1	72.7	73.6	21.9
Ratio R/U	**1.5**	**1.7**	**1.7**	**2.3**

N/A: not available.

With a slight difference between urban and rural areas, the use of modern contraceptive methods increased from 62.8% in 1992 to 67.4% in 2011.

According to the Ministry of Health, the improvement in women’s access to health care contributed to the significant reduction in the maternal mortality ratio (MMR) from 227 in 2004 to 112 in 2010 [6].

In the 2011 survey sample anthropometric measures were available for a total of 7310 children under five years of age. A child was considered as underweight if his or her weight-to-age ratio was < 2 SD the median weight-to-age ratio of the sample. Similarly, children were considered stunted (respectively wasted) if their height-to-age ratio (respectively, weight-to-height ration) was < 2 SD the median of the sample. The proportion of stunted children decreased constantly between 1992 (22.6%) and 2011 (14.9%), while the proportions of underweight and wasted children increased between 1992 and 2004 and then decreased between 2004 and 2011 (Table 5).

**Table 5:** **Urban/rural differences in Moroccan child nutrition according to the last three National Surveys on Population and Health 1992, 2004 and 2011 **[2,3]

	**Stunting (%)**	**Underweight (%)**	**Wasting (%)**
Year 1992			
Urban	13.0	1.9	3.3
Rural	27.0	2.4	12.0
National	22.6	2.3	9.0
Ratio Rural/Urban	**2.1**	**1.3**	**3.6**
Year 2004			
Urban	12.9	6.5	7.6
Rural	23.6	14.1	11.1
National	18.1	9.3	10.2
Ratio Rural/Urban	**1.8**	**2.2**	**1.5**
Year 2011			
Urban	8.6	1.7	1.6
Rural	20.5	4.3	3.0
National	14.9	3.1	2.3
Ratio Rural/Urban	**2.4**	**2.5**	**1.9**

The infant mortality rate decreased from 63 in 1992 to 33.6 in 2011. Similarly, the under-five mortality rate decreased from 84 to 35 during the same period of time. Neonatal mortality decreased from 34 in 1992 to 20.5 in 2011.

## Discussion

While in 1950 nearly 71% of the world population was living in rural areas, in 2008, half of the world population was living, for the first time in history, in urban areas. If the present trend of transition is maintained, it is expected that 70% of the world population will be living in urban areas by 2050. Worldwide, average urban incomes are generally higher than those in rural areas. Urban dwellers also have better access to a variety of services, including education, health, transportation, communication, water supply, sanitation and waste management. For policy makers, it is more efficient and cheaper to provide such services to large and geographically concentrated populations than to populations scattered over large rural areas [7]. In Morocco, 71% of the population lived in rural areas in 1960, about 50% in 1990 and 42% in 2011. The Moroccan transition can also be explained by a large rural exodus due to scarcity of water and the decline of agriculture which used to be the main economic activity for rural dwellers.

The age structure of the Moroccan population has changed over the last three decades. Between 1980 and 2011, the young category (< 15 years) decreased from 43.6% to 29% of the total population, while the percentage of older persons (> 65 years) in the total population grew from 3.7% to 6.8%.

Between 1960 and 2011, the mean age of first marriage increased by 6.5 years for men and 4.8 years for women. This delay can be explained by a multitude of cultural and socio-economic factors such as the access of young girls to higher education, unemployment, housing problems, the high expenses of marriage for men (responsible for the dowry and the cost of the wedding ceremony and associated festivities) and the high cost of bringing up children [8].

The total fertility rate, defined as the number of children who would be born to a woman if she were to live to the end of her childbearing years, decreased by three children during the last three decades. The fertility decline is mainly attributable to factors such as the use of contraception and the delayed age of marriage. For instance, between 1980 and 2011, the proportion of married women using contraception increased from 19% to 67.4%. During the same period of time, the proportion of married women in the cohort aged 20–24 declined from 64% to 12%. Low fertility can also be explained, however, by socio-economic factors such as access of girls to higher education, unemployment and the high cost of maintaining a decent living standard (food, housing, education, health care, transport, leisure, etc.) [8-10].

Despite the efforts of the Moroccan authorities to reduce illiteracy, 37.6% of the population aged 10 years and over was illiterate in 2011. Moreover, rural populations are twice as likely to be illiterate (53.6%) compared to their urban counterparts (25.4%). The rate of illiteracy among women (48.4%) is greater than for men (26%). For the age category 15–24 years, 38% of rural females are illiterate compared to 4% of urban males, a ratio of 9.27. The comparison between the1992 and 2011 data shows that the gender gap as well as the relative ratio between urban and rural areas are persisting or even increasing.

Unemployment data also show large gaps since only 6.4% of women in rural areas (respectively 18.6% in urban areas) had a job in 2011, compared to 76.2% of rural men (respectively 62.8% of urban men).

During the last decade, noticeable efforts were devoted by Moroccan health authorities to maternal and infant health in order to achieve the Millennium Development Goals 4 and 5, which stipulated the reduction of the child mortality rate and the maternal mortality ratio by two-thirds and three-quarters respectively between 1990 and 2015. In 2011, four years ahead of the deadlines, the results obtained are satisfactory and Morocco is on track to achieve these two MDGs [6,11,12]. While applauding this achievement as an average, one needs also to assess the strategy in terms of inequality reduction. According to the 2011 survey, rural–urban gaps are persisting in terms of access to health care and services. For instance, more than 90% of urban women deliver in health facilities with the assistance of skilled health personnel, compared to 55% for rural women. A more profound gap is seen in access to postnatal care, accessed by only 13.2% of rural women compared to 30.5% of urban women (Table 4).

Neonatal, postnatal, infant and under-five mortality all decreased significantly between 1992 and 2011. For instance, infant mortality rate was more than halved, decreasing from 63 to 29. The urban–rural gaps remained globally at the same level (Table 5), however.

Finally, the 2011 survey showed that, although the mean proportions of stunting, underweight and wasted children decreased between 1992 and 2011, the gaps between the rural and urban population for these indictors all increased during the period 2004-2011 (Table 6).

**Table 6:** **Infant mortality and rural/urban distribution of the Moroccan population participating in the last three National Surveys on Population and Health 1992, 2004 and 2011 **[2,3]

	**Neonatal mortality rate**	**Postnatal mortality rate**	**Infant mortality rate**	**Under 5 mortality rate**
Year 1992				
Urban	30.0	22.0	52.0	59.0
Rural	36.0	33.0	69.0	98.0
National	34.0	29.0	63.0	84.0
Ratio R/U	**1.2**	**1.5**	**1.3**	**1.7**
Year 2004				
Urban	24.0	9.0	33.0	38.0
Rural	33.0	22.0	55.0	69.0
…….National	27.0	14.0	40.0	47.0
Ratio R/U	**1.4**	**2.4**	**1.7**	**1.8**
Year 2011				
Urban	17.0	6.6	23.6	25.4
Rural	20.5	13.0	33.6	35.1
National	18.9	10.0	28.9	30.5
Ratio R/U	**1.2**	**2.0**	**1.4**	**1.4**

### Strength and limitation of the study

Beyond the evolution of health and socio-economic indicators as national averages, it is necessary to monitor inequity health indicators. Consequently, data provided by national surveys at regular intervals of time constitute valuable resources for analysis and comparisons. The 2011 survey was needed in order to update health and socio-economic indicators which are indispensable for the development of optimal policies and efficient and effective strategies. Up to the present time, however, the Moroccan Ministry of Health has not provided access to the whole survey data base. The limited available data were used to show that inequalities must be reduced alongside the improvement of socio-economic indicators as averages. Information from the 2011 survey can help policy makers to mitigate disparities at the regional level, as well as differences in mother’s education and the socio-economic status of the household.

## Conclusion

Moroccan authorities carry out regular surveys on population and family health (NSFFP 1980, NSPH 1992, SPFH 2004, NSPFH 2011) [2,3]. These surveys constitute valuable resources for monitoring socio-economic and health status. They provide health and social indicators to assist health decision makers to develop effective policies and equitable strategies.

In this paper, data provided by the recent NSPFH 2011 survey are compared with those from two previous surveys, NSPH 1992 and SPFH 2004. This analysis of demographic and socio-economic indicators gives special attention to maternal and infant health indicators, showing that, as national averages, health indicators have improved noticeably during the last two decades, but that rural–urban disparity and gender inequality in literacy and employment are still challenging health decision makers. Therefore Moroccan authorities need to make serious efforts to reduce health and socio-economic inequalities as well as paying attention to improving national indicators measured as averages. This concern for equitable outcomes follows the recommendations of the World Health Organization Commission on Social Determinants of Health [4], the recent United Nations “Common Country Assessment Report” [11] and the UNICEF objective of “narrowing the gaps to meet the goals” [12]. It highlights the problem that strategies based on national average indicators like MMR, IMR, life expectancy and the MDGS may reach their stated goals, but at the same time the gaps between men and women, rich and poor, rural and urban, developed and less developed regions may persist or even increase.

## Competing interests

The author declares that he has no competing interest
